# Bone marrow-derived vasculogenesis leads to scarless regeneration in deep wounds with periosteal defects

**DOI:** 10.1038/s41598-022-24957-1

**Published:** 2022-11-29

**Authors:** Yuuki Shirai, Junko Okano, Takahiko Nakagawa, Miwako Katagi, Yuki Nakae, Atsuhiro Arakawa, Shinya Koshinuma, Gaku Yamamoto, Hideto Kojima

**Affiliations:** 1 Department of Oral and Maxillofacial Surgery, Shiga University of Medical Science, Shiga, Japan; 2 Department of Plastic and Reconstructive Surgery, Shiga University of Medical Science, Shiga, Japan; 3 Department of Regenerative Medicine Development, Shiga University of Medical Science, Shiga, Japan; 4 Department of Biocommunication Development, Shiga University of Medical Science, Shiga, Japan; 5 Department of Stem Cell Biology and Regenerative Medicine, Shiga University of Medical Science, Shiga, Japan

**Keywords:** Biochemistry, Immunochemistry, Stem cells, Regeneration, Stem-cell niche, Skin diseases, Trauma

## Abstract

Deep skin wounds with periosteal defects, frequently caused by traffic accidents or radical dissection, are refractory. Transplant surgery is frequently performed, but patients are subjected to stress for long operation periods, the sacrifice of donor regions, or several complications, such as flap necrosis or intractable ulcers. Even if the defects are covered, a scar composed of fibrous tissue remains in the body, which can cause itching, dysesthesia, or repeated ulcers because of the lack of distribution of peripheral nerves or hair follicles. Thus, treatments with the aim of regenerating lost tissue for deep wounds with periosteal defects are needed. Here, we show that the use of gelatin sponges (GS), which have been used as haemostatic materials in clinical practice, allowed the regeneration of heterogeneous tissues, including periosteum, skin, and skin appendages, when used as scaffolds in deep wounds with periosteal defects in rats. Bone marrow transplantation in rats revealed the mechanism by which the microenvironment provided by GS enabled bone marrow-derived cells (BMDCs) to form a vascular niche, followed by regeneration of the periosteum, skin, or skin appendages such as hair follicles by local cells. Our findings demonstrated that vascular niche formation provided by BMDCs is crucial for heterogeneous tissue regeneration.

## Introduction

Wounds never heal with regenerated tissue but fibrous tissue after birth^1–3^, although in a foetus, lost tissues completely regenerate, which we call “scarless wound healing”^4^. Thus, in the clinical setting, the goal of patients with deep wounds remains epithelization with fibrous tissue, “scar”^5^. Because deep full-thickness skin wounds with periosteal defects have risks such as deep infection followed by osteomyelitis and sepsis shock in patients with underlying diseases such as diabetes and/or advanced age, transplant surgery utilizing either fasciocutaneous or musculocutaneous flaps is performed^6^. However, even if the surgery is successful, patients are subjected to stress for scarring because not only skin appendages such as hair follicles (Hfs) and nerves but also layered structures such as the periosteum and skin are lost, which causes itching, dysesthesia, or repeated ulcers^7,8^. To solve such problems, a combination of conventional flaps and periosteal flaps has been developed, since the periosteum is a source of stem cells as well as numerous growth factors for bone formation^9^. However, this method is not as prevalent as expected, probably because the procedure is not only complicated but also causes more donor sacrifices^10^. Therefore, nonsurgical treatments are needed to enable regeneration of lost tissue in deep wounds with periosteal defects.

Treatments using scaffolds for wounds could be alternative treatments. Indeed, the application of scaffolds could be efficient^11–15^ for deep full-thickness skin wounds alone, although the goal of contemporary treatments using scaffolds is to accelerate wound healing by promoting scar formation^16,17^. In fact, novel treatments for wounds with periosteal defects with the aim of regenerating lost tissue but not forming scars are challenging.

In the present study, we developed tissue regeneration for deep wounds with periosteal defects utilizing scaffolds. We demonstrated that the use of a gelatin sponge (GS) allowed scarless healing in the exposed calvaria bone in rats. Surprisingly, GS in deep wounds with periosteal defects enabled vasculogenesis promoted by bone marrow-derived cells (BMDCs), leading to heterogeneous regeneration, including in the periosteum, skin, and skin appendages.

## Results

### GS provides microenvironments adequate for tissue regeneration in deep wounds with periosteal defects

**Figure 1:**
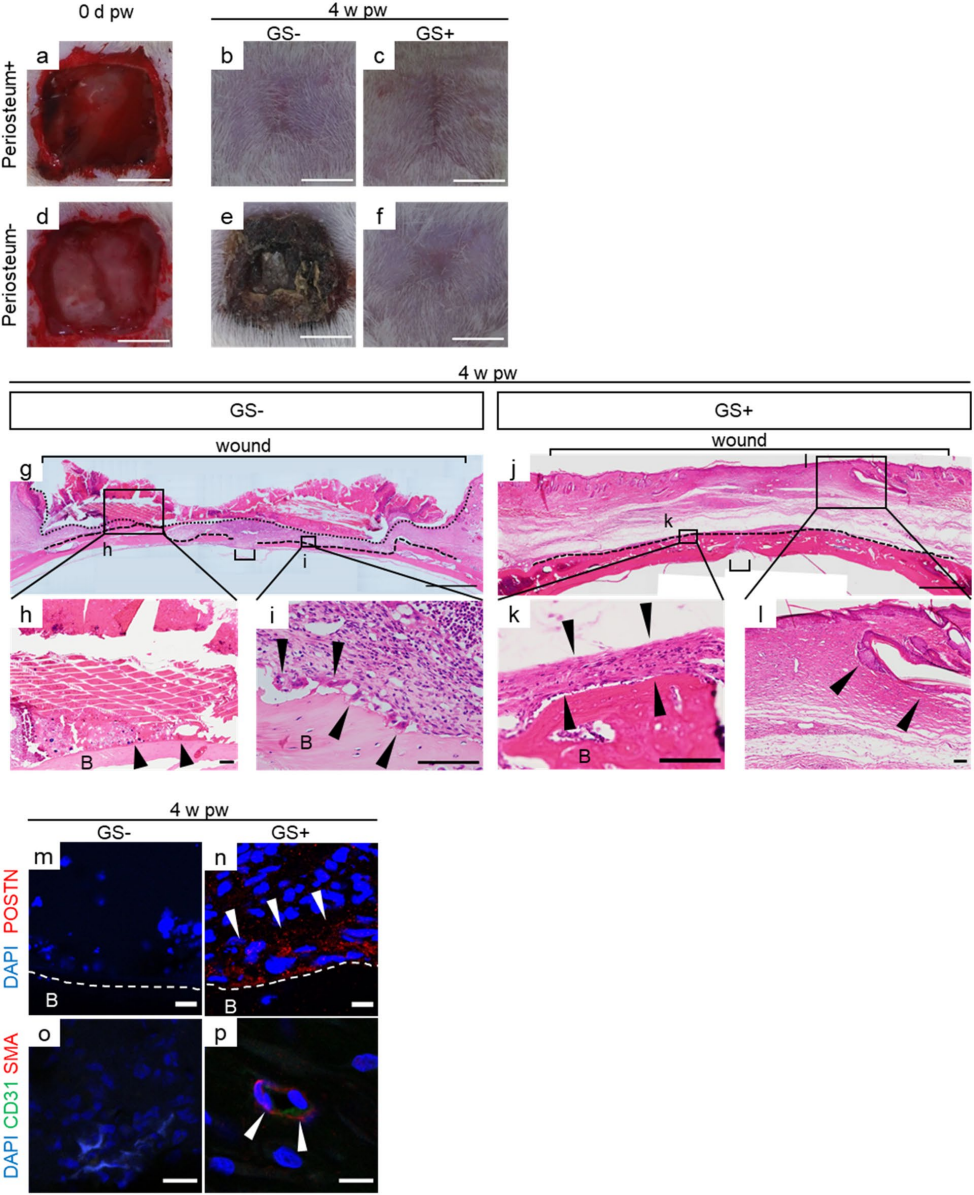
GS allows regeneration of heterogeneous tissues in deep wounds with exposed bone cortex. (**a**–**f**) Gross appearance of skin/subcutaneous defects with/without periosteum defects when GS is applied or not is shown after manipulation (0 d pw) and at 4 weeks pw. (**g**–**l**) H&E staining of deep wounds with periosteal defects at 4 pw is shown. A high-power view reveals the invasion of immune cells into the bone cortex in open wounds (**h**,**i**, arrowheads), while membranous structures and hair follicles are present in GS-treated wounds (**k**,**l** arrowheads). (**m**–**p**) Periostin (red), a marker of the periosteum, is positive in membranous structures and cavity structures with smooth muscle actin (red), and CD31 positivity (green) is present in the GS-treated wounds. n = 4 for each. The experiments were repeated twice. Scale bars, 5 mm in (**a**–**f**), 1 mm in (**g**, **j**), 100 μm in (**h, i, k, l**), 10 μm in (**m**–**p**). *d* day, *w* week, *pw* post-wounding, *GS*− open wound, *GS*+ GS-treated wound, *B* bone. Wound with brackets in (**g**) and (**j**), the regions excised at the beginning; dotted line, the apical boundary; dashed lines, the surface of bone cortex; *DAPI* 4′6-diamidino-2-phenylindone, *POSTIN* periostin, *SMA* smooth muscle actin.

We compared the process of wound healing in our model using rats in the case of skin and subcutaneous tissue defects with or without periosteal defects. First, deep wounds without periosteal defects were made in the calvaria in rats (Fig. 1a). The wounds were left open in one group, while GS was applied to the wounds in the other group (Fig. 1b,c). As a result, wounds were healed with sparse hair follicles (Hfs) at 4 weeks post-wounding (4 w pw hereafter), regardless of GS application (Fig. 1b,c). This result suggests that the process of wound healing is independent of the use of scaffolds in the case of skin and subcutaneous tissue defects in our model.

Next, the same series of wounds, including excision of the periosteum, were made in the calvaria of rats to recapitulate deep wounds with periosteal defects in clinical practice (Fig. 1d). Then, wounds were harvested in one group (GS (−) rats, thereafter), while GS was applied to the wounds in the other (GS (+) rats, thereafter). At 4 w pw, the defect was encrusted in the calvaria in GS (−) rats, whereas GS application allowed similar wound healing to that in the case of wounds with periosteum (Fig. 1b,c,e,f). H&E staining showed that bone cortex defects were observed in the centre of a wound, possibly due to osteolysis (Fig. 1g, bracket), which was surrounded by a thinner bone cortex in GS (−) rats than in GS (+) rats (Fig. 1g,j, dashed lines). A high-power view revealed that there were immune cells and debris adjacent to bone and that multinuclear cells, probably osteoclasts, invaded the bone cortex, suggesting inflammation because of osteolysis in GS (−) rats (Fig. 1h, i, arrowheads). In contrast, in GS (+) rats, the thickness of bone in the centre of a wound was comparable to that of the surrounding bone (Fig. 1j, bracket), and surprisingly, the exposed bone was covered with membranous tissue (Fig. 1k, arrowheads). In addition, Hfs were observed in wounds (Fig. 1l, arrowheads). We explored the membranous tissue covering the bone cortex at 4 w pw in GS (+) rats and found that it was positive for periostin, which was preferentially expressed in the periosteum, although it was not present in the bone of the wounds in GS (−) rats^18^ (Fig. 1m,n). In turn, we investigated whether vasculogenesis would occur in the newly formed skin in deep wounds with periosteal defects because recent research has shown that local vasculogenesis allows tissue regeneration^19^. As a result, the structures of vessels with CD31- and smooth muscle actin (SMA)-positive were observed in neo-dermis GS (+) rats at 4 w pw, although they were not in GS (−) rats (Fig. 1o,p). Specifically, CD31-positive cells were surrounded by SMA-positive cells (Fig. 1p). Taking into consideration that CD31 is an endothelial cell marker and SMA is expressed in vascular smooth muscle cells of arterioles^20,21^, the observed vessels in the neo-dermis appeared functional in GS (+) rats.

Taken together, GS application enabled tissue regeneration of the periosteum as well as skin tissue in deep wounds with periosteal defects in rats.

### The healing process of deep wounds with periosteal defects in BMT rats mimicked wound healing in wild rats

**Figure 2:**
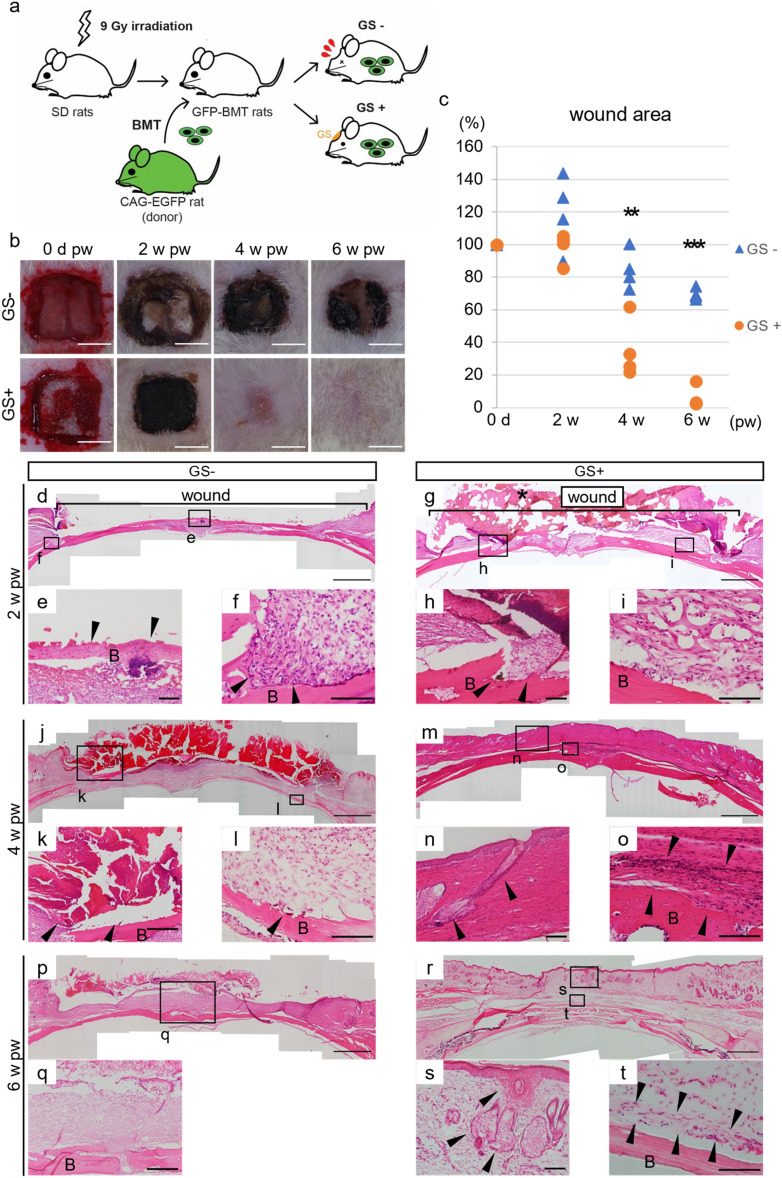
Heterogeneous tissue regeneration by GS application occurs in deep wounds with periosteal defects in a BMT rat model. (**a**) Schema of establishing BMT rats. After 9 Gy irradiation exposure, 1 × 10^8^ bone marrow cells isolated from green fluorescent protein (GFP) rats were injected into the tail vein of recipient rats. One month later, skin/subcutaneous tissue/periosteum defects were made in the calvaria with/without GS application. n = 4 for each. (**b**) Gross appearance of the deep wounds with or without GS application is shown at various time points. (**c**) Wound area with or without GS application was quantified. (**d**–**t**) H and E staining of the wounds with or without GS application are shown. The experiments were repeated twice. Scale bars, 5 mm in c, 1 mm in (**d**,**g**,**j**,**m**,**p**,**t**) 100 μm in (**e**,**f**,**h**,**i**,**k**,**l**,**n**,**o**,**r**,**u**,**v**). Arrowheads, see the text. *B* bone cortex. *P < 0.05, **P < 0.01, ***P < 0.005.

Our group has clarified the critical roles of BMDCs in various mouse models, such as diseases, trauma, or physical phenomena, neuropathy or renal failure caused by diabetes, the healing process from bone fractures, or appetite^22–25^. Therefore, we asked whether BMDCs are associated with tissue regeneration by GS application in deep wounds with periosteal defects in rats. With this aim, we developed bone marrow transplantation (BMT) rats receiving allogenic BM cells from GFP rats after 9 Gy irradiation, followed by making deep wounds, including periosteum excision, and dividing them into two groups, i.e., GS (−) and GS (+) rats (Fig. 2a). Since exposure to the dose of irradiation necessary for eliminating BM cells in recipient rats causes skin dysfunction in mice^26^, we examined whether GS application would allow tissue regeneration in deep wounds with periosteal defects in BMT rats.

One month after BMT, chimerism analysis confirmed that more than 87% of peripheral blood cells were of donor origin (Supplemental Table 1). Despite skin exposure to irradiation at moderate doses, a gross appearance showed that a similar process was observed in GS (+) BMT rats to that in GS (+) rats, as shown in Fig. 1, although crusts remained attached to the wounds at 6 w pw in GS (−) BMT rats (Fig. 2b). The time-course analyses of wound areas showed significantly decreased wound areas in GS (+) BMT rats compared to those in GS (−) BMT rats at any evaluated points (Fig. 2c). H&E staining did not show a significant difference between GS (−) BMT and GS (+) BMT rats in lower magnification images at 2 w pw, except that GS degradation had already started in GS (+) BMT rats (Fig. 2d,g). However, high magnification images revealed that osteolysis had already developed in the centre of the exposed bone cortex and that invasion of osteoclast-like cells into the bone cortex was observed at the edge of the wound in GS (−) BMT rats at 2 w pw (Fig. 2e,f). Conversely, not only was such cell invasion into the bone cortex observed at the edge of the wound, but numerous cavities surrounded by cells were also observed in GS (+) BMT rats at 2 w pw (Fig. 2h,i). At 4 w pw, similar images were obtained, as shown in Fig. 1g–l, which suggested that BMT rats recapitulated normal wound healing (Fig. 2j–o). At 6 w pw, fibrous tissue was observed on the bone cortex in GS (−) BMT rats, but surprisingly, the layered skin was reconstructed with even de novo Hfs/sebaceous glands in the centre of the wound in GS (+) BMT rats (Fig. 2p–s). In addition, the accumulation of cells on the bone cortex suggested the regeneration of the periosteum (Fig. 2t).

Altogether, our data suggest that the wound healing process was similar in BMT rats and wild rats despite skin exposure to moderate doses of irradiation. Consequently, we analysed the process of wound healing of deep wounds with periosteal defects with/without GS utilizing BMT rats.

### GS allowed BMDC involvement during vasculogenesis

**Figure 3:**
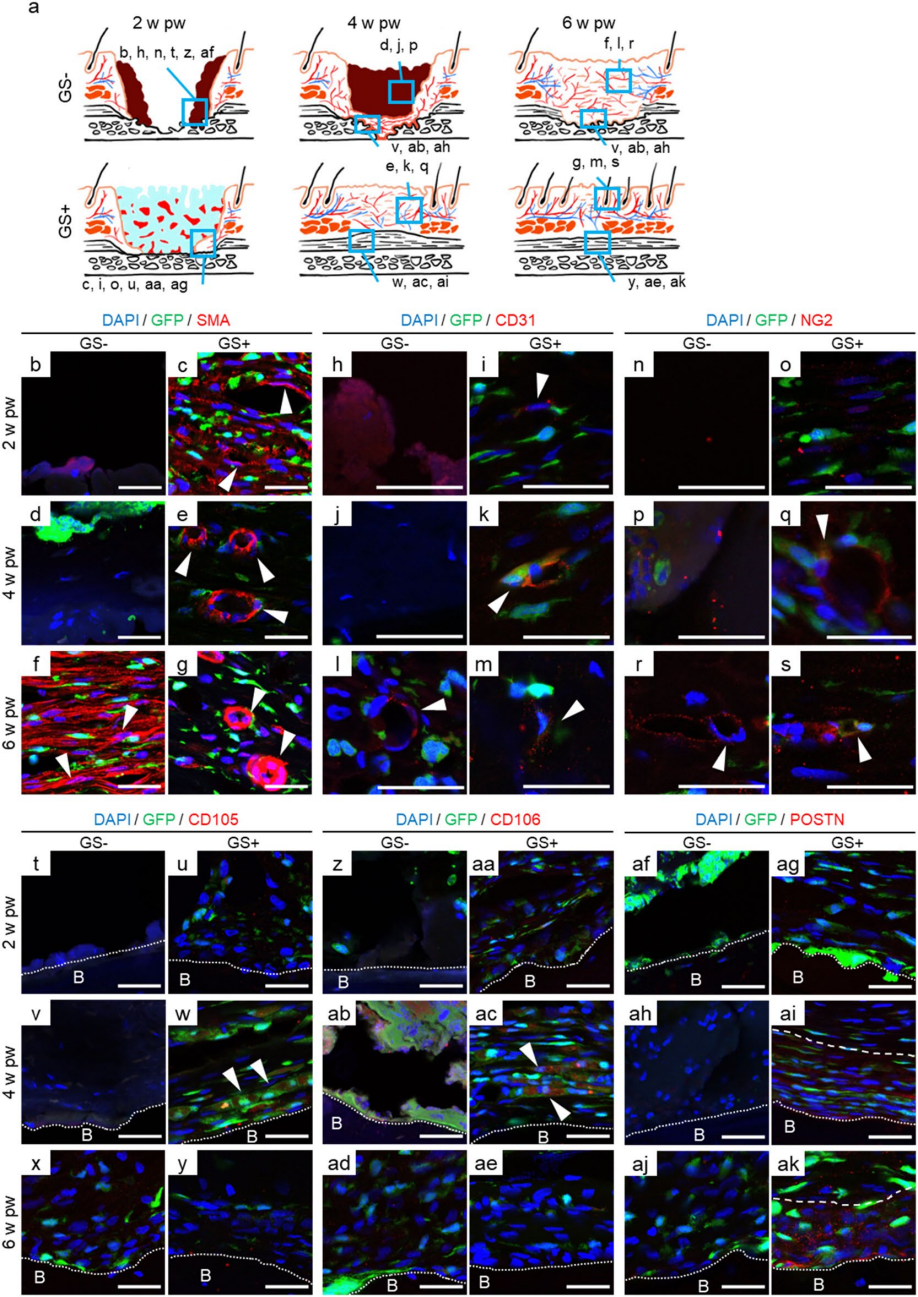
Vasculogenesis in heterogeneous tissues led by GS application in BMT rats. (**a**) Schematic drawings of the deep wounds with or without GS application is shown at various time points. (**b**–**s**) Neovessel formation in the dermis of GS (+) rats. (**b**–**g**) Immunohistochemistry with anti-SMA antibody (red) reveals luminal structures contributed by BMDCs (green) in GS (+) rats, whereas SMA-positive BMDCs exhibit fibre-like patterns at 6 w pw in GS (−) rats. (**h**–**m**) CD31 stains BMDCs in luminal structures in GS (+) rats, which is never present in GS (−) rats. (**n**–**s**) NG2, a periderm marker (red), is positive for BMDCs surrounding newly formed endothelial cells in GS (+) rats but not in GS (−) rats. (**t**–**ae**) Vasculogenesis occurs in GS adjacent to bone. CD105 and CD106, markers of vascular endothelium, are stained in BMDCs at 4 w pw in GS (+) rats (arrowheads in **v** and **ab**). (**af**–**ak**) Periostin is positive in the tissue adjacent to bone after 4 w pw in GS (+) rats (between dot line and dash line in **ah** and **aj**) but not present in GS (−) rats. n = 4 for each. The experiments were repeated twice. Scale bars, 30 μm. Dotted lines show the boundary of the bone cortex. The dashed line shows the apical boundary of the periosteum. *B* bone.

**Figure 4:**
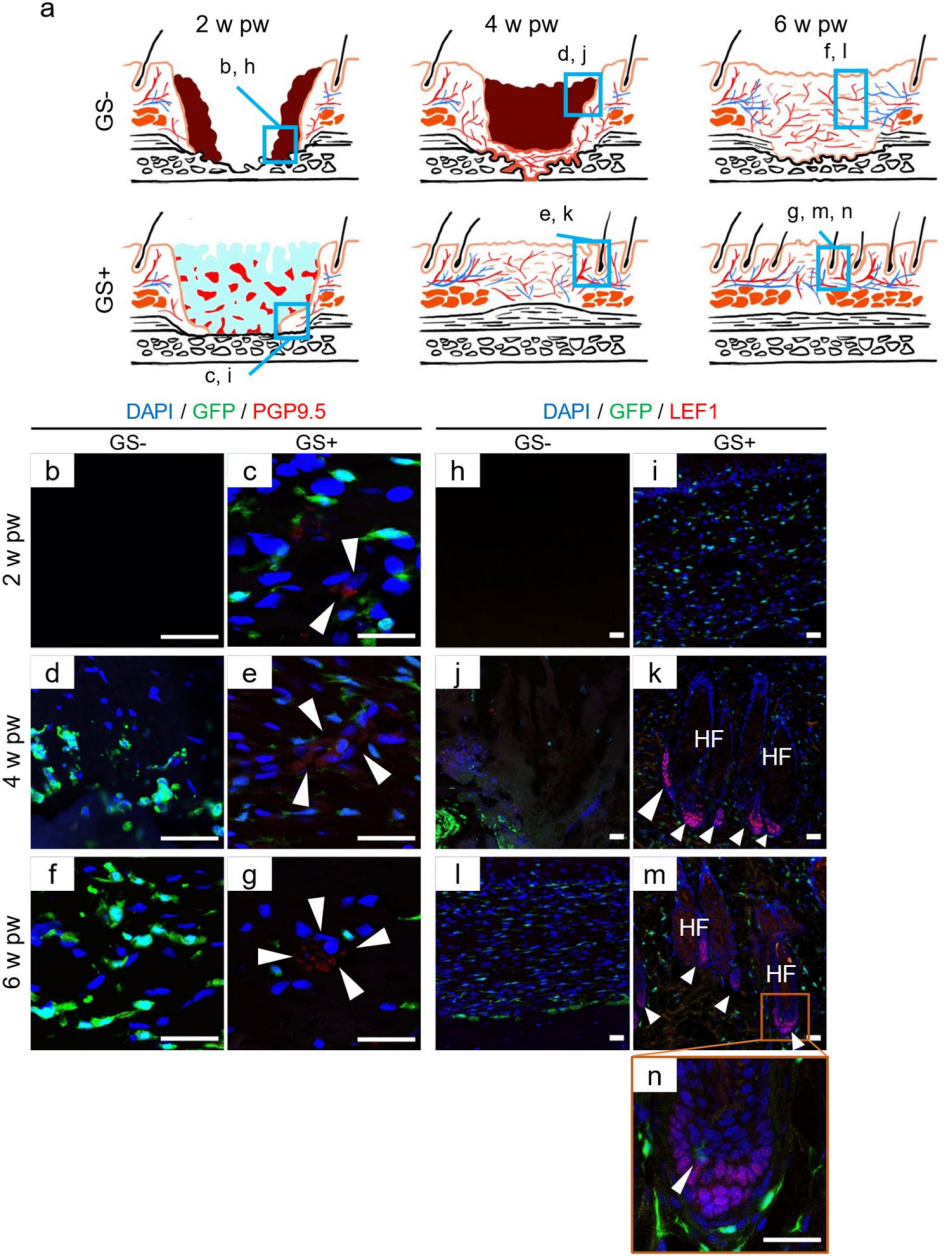
GS allows regeneration of nerves and appendages of the skin. (**a**) Schematic drawings of the deep wounds with or without GS application is shown at various time points. (**b**–**g**) PGP 9.5 (red)-positive cells (arrowheads) are scattered at 2 w pw and commence to form assemblies after 4 w pw in GS (+) rats but not in GS (−) rats. (**h**–**n**) Lef1 (red) is positive in the dermal papilla of de novo hair follicles (arrowheads) after 4 w pw in GS (+) rats but not in GS (−) rats. (**n**) High-power view showing that BMDCs are Lef1-negative. n = 4 for each. The experiments were repeated twice. *HF* hair follicles, Scale bars, 30 μm.

During the process of wound healing, an inflammation phase is replaced with a resolution phase for tissue regeneration^27^. The first step of a resolution phase is vasculogenesis, and the skin is supplied by arterioles, venules and capillary loops that connect with arterioles and venules in the dermis^28^. Thus, we examined vasculogenesis in wounds with periosteal defects utilizing BMT rats (Fig. 3a). Immunohistochemistry using anti-alpha smooth muscle actin (αSMA), which is abundantly expressed in arterioles, showed a widely scattered pattern in the entire tissue in degraded GS, and interestingly, part of the luminal structure-like pattern with SMA-positive cells was frequently observed at 2 w pw in GS (+) rats, while almost no cell population was present in GS (−) rats (Fig. 3b,c). Notably, structures such as lumina were surrounded by BMDCs (GFP-positive cells) in GS (+) rats (Fig. 3c). At 4 w pw, BMDCs with SMA-positive cells exhibited obvious luminal structures, although SMA expression was not observed in GS (−) rats (Fig. 3d,e). In turn, the wall of arterioles with SMA/GFP-positive cells became thicker at 6 w pw than 4 w pw in GS (+) rats (Fig. 3g). Conversely, in GS (−) rats, strong SMA-positive BMDCs exhibited fibre-like patterns, indicating fibrotic change, namely, a “scar” at 6 w pw^29,30^ (Fig. 3f). Next, CD31, which is known as an endothelial cell adhesion molecule and allows arteriole, venule and capillary loops to be visualized, was examined, and it was found that several BMDCs commenced to express CD31 at 2 w pw in GS (+) rats but not in GS (−) rats (Fig. 3h,i). BMDCs with CD31 positivity were observed to form luminal structures at 4 w pw as well as 6 w pw in GS (+) rats (Fig. 3k,m). In contrast, luminal structures with CD31 positivity were not observed at 4 w pw but at 6 w pw in GS (−) rats, although BMDCs did not contribute to those structures (Fig. 3j,l). Capillary loops are lined by endothelial cells, outside of which pericytes are present, which are contractile cells^20^. Therefore, NG2 expression, a pericyte marker, was stained to examine how GS application affected vasculogenesis. Consequently, NG2-positive cells were observed with a scattered pattern as with SMA staining at 2 w pw, accompanied by NG2-positive BMDCs, which formed luminal structures at 4 w pw in GS (+) rats, although such patterns were not observed in GS (−) rats (Fig. 4n–q). At 6 w pw, luminal structures lining NG2-positive staining were sporadically present in GS (−) rats, which were GFP-negative, whereas notably, both GFP- and NG2-positive cells surrounded luminal structures in GS (+) rats (Fig. 3r,s).

Subsequently, CD105 and CD 106, markers of vascular endothelium, were examined to explore vasculogenesis in the bone surface after wounding. At 2 w pw, there were few cells on the exposed bone cortex in GS (−) rats, whereas numerous cells, including BMDCs, accumulated on the bone cortex in GS (+) rats (Fig. 3t,u,z,aa). At that time, periostin was not expressed in either GS (−) or GS (+) rats (Fig. 3af,ag). At 4 w pw, CD105- or CD106-positive cells started to be observed, which were also GFP-positive, respectively, in GS (+) rats (Fig. 3w,ac). At 6w pw, neither CD105- or CD106-positive cells were present in GS (+) rats (Fig. 3y,ae). Simultaneously, periostin was observed in GS (+) rats at 4 w pw, becoming evident at 6 w pw (Fig. 3ai,ak). In turn, neither expression of CD105 nor CD106 was present at 4 w and 6 w pw, although local cells and BMDCs that accumulated at 6 w pw failed to express periostin in GS (−) rats (Fig. 3v,x,ab,ad,ah,aj). Quantitative analyses revealed that BMDCs significantly contributed to the regeneration of the vessels and the periosteum in GS (+) rats (Supplemental Table 2).

Taken together, these data suggest that BMDCs play an essential role in vasculogenesis, which was made possible by GS application.

### Nerves and appendages of the skin are regenerated by local cells in GS (+) rats

We investigated the regeneration of micro-organs in wounds with periosteal defects (Fig. 4a). The skin is innervated by cutaneous small nerve fibres to transmit heat, pain, and other sensations^31,32^. The expression of PGP9.5, a pan axonal maker, was not observed in GS (−) rats (Fig. 4b,d,f). In contrast, it started to be expressed at 2 w and 4 w pw, followed by organized assemblies by GFP-negative cells at 6 w pw in GS (+) rats (Fig. 4c,e,g, arrowheads). In turn, the development of de novo Hfs was examined using an anti-Lef1 antibody because Lef1 was expressed in hair germ during HF development, contributing to the differentiation of bulge stem cells^33^. We found that Lef-1 was expressed in the dermal papilla of de novo Hfs, as in that of Hfs around wounds, composed of GFP-negative cells after 4 w pw in GS (+) rats (Fig. 4i,k,m, arrowheads and Supplemental Fig. 1). High-power views revealed that a few GFP-positive cells were present in de novo Hfs, which were Lef1-negative (Fig. 4n, arrowhead). On the other hand, no Lef-1 expression was compatible with no Hfs in the wounds of GS (−) rats (Fig. 4h,j,l). Collectively, GS application in wounds with periosteal defects allowed the regeneration of skin appendages by local cells.

### Dynamic change in the regenerating process in GS (+) rats

**Figure 5:**
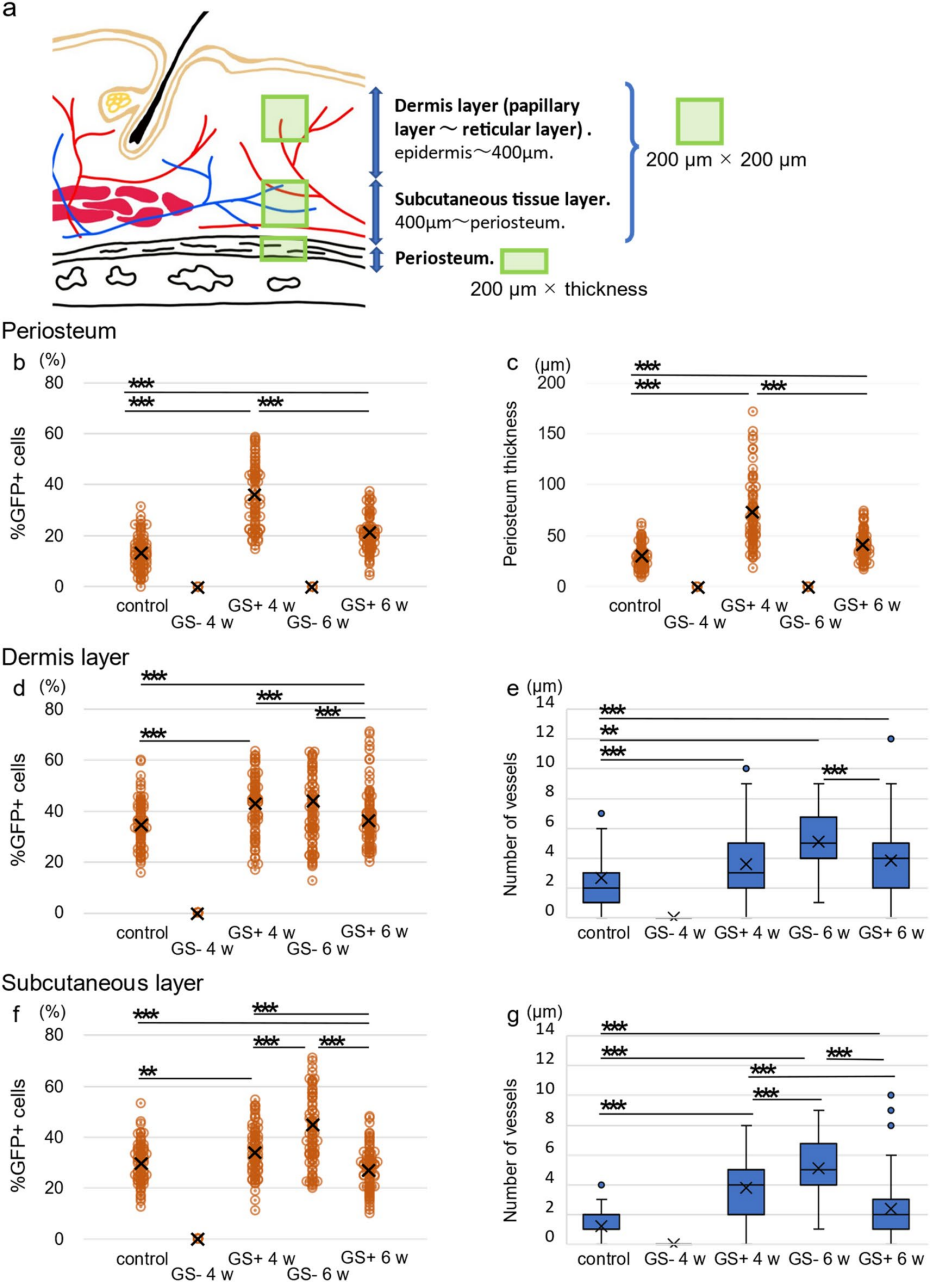
Quantitative analyses in regenerating tissue due to GS application. (**a**) Schema of the tissues in the rat calvaria. The dermis is defined as the tissue from the epidermis–dermis junction to a depth of 400 μm, and from there to the periosteum, it is further defined as subcutaneous tissue. The green areas of either squares or rectangles were analysed. (**b**,**c**) The changes in the rates of GFP migration into the periosteum and the periosteal thickness in control (unwounded healthy BMT rats), GS (−), and GS (+) rats at 4 w and 6 w pw. (**d**,**e**) The changes in the rates of GFP migration into subcutaneous tissues and the numbers of vessels in control, GS (−), and GS (+) rats at 4 w and 6 w pw. (**f**,**g**) Changes in the rates of GFP migration into the dermis and the numbers of vessels in control, GS (−), and GS (+) rats at 4 w and 6 w pw. The cross of each scatter or box plot indicates average value. The upper or lower whisker of each box plot indicates maximal or minimal value, respectively. n = 4 for each. The experiments were repeated twice. *P* < 0.05, **P < 0.01, ***P < 0.005.

Finally, we investigated the roles of BMDCs in the periosteum and skin in wounds with periosteal defects. To address this issue, the percentage of migrating BMDCs was analysed in each tissue, and regenerated structures, such as the periosteum and the vessels, were quantified at 4 w and 6 w pw (Fig. 5a). We also analysed the unwounded (healthy) skin and periosteum in the calvaria in BMT rats (control group), which were regarded as steady state.

The percentage of GFP+ cells in the periosteum peaked at 4 w pw and decreased at 6 w pw in GS (+) rats (Fig. 5b). There were no GFP+ cells at the two timepoints in the periosteum of GS (−) rats (Fig. 5b). Notably, substantial GFP+ cells were observed in unwounded skin, whereas the percentage of GFP+ cells in GS (+) rats at 6 w pw was significantly larger than that in unwounded rats (Fig. 5b). This tendency was also the case with the thickness of the regenerated periosteum, i.e., the periosteum in GS (+) rats was thicker at 4 w pw than at 6 w pw, although the periosteum in GS (+) rats at 6 w pw was thicker than that in unwounded rats (Fig. 5c). Such differences between the condition under unwounded periosteum and GS (+) rats at 6 w pw appeared the status in which the regeneration was still in processing or the limitation of GS as the use of scaffolds used in our study.

Interestingly, the percentage of GFP+ cells migrating into the dermis or subcutaneous tissue was different from that in the periosteum. Hence, the percentage of GFP+ cells migrating into the dermis and subcutaneous tissue peaked at 4 w pw as that in the periosteum in GS (+) rats, while it was similar at 6 w pw in GS (+) rats to that in unwounded dermis or subcutaneous tissue (Fig. 5d,f). In addition, more GFP+ cells migrated in the dermis and subcutaneous tissue in GS (−) rats than in GS (+) rats at 6 w pw (Fig. 5d,f). The numbers of vessels in dermis or subcutaneous tissue correlated positively with the results of the percentage of migrating GFP+ cells, suggesting that BMDCs play essential roles in vasculogenesis in regenerated tissue (Fig. 5e,g). These results suggest the following: (i) the condition of subcutaneous tissue as well as dermis in GS (+) rats at 6 w pw appear comparable to that of unwounded tissue, unlike periosteum, (ii) in GS (−) rats, the peak of the percentage of migrating GFP+ cells into dermis and subcutaneous tissue and the numbers of vessels was delayed.

Altogether, the microenvironments that GS provided to deep wounds with periosteal defects made it possible for the dynamic and efficient migration of BMDCs into wounds to accomplish regeneration, while BMDCs were shown to be supplied to local tissues even under a steady state.

## Discussion

In the present study, we determined that the application of GS in deep wounds with periosteal defects allowed BMDCs to differentiate into pericytes, endothelial cells, or vascular smooth muscle cells which compose vessels, leading to regeneration of the periosteum as well as skin with appendages and nerves in the calvaria of rats. Indeed, there are several studies in which GS and collagen sponges are used for scaffolds in bone defect models, but most of them showed that the use of scaffolds facilitated wound healing, not heterogeneous tissue regeneration, as suggested by our study^34–36^. The mechanism of facilitated wound healing has been known that the application of scaffold in wounds allowed local cells to migrate into wounds^37^. Notably, we demonstrated that GS application provided the microenvironments adequate for the migration of BMDCs as well as local cells into the wounds at early stage, resulting in vasculogenesis crucial for tissue regeneration. One of the important factors to allow BMDCs migration might be pore size, 100 mm on average, which is relatively large compared with other materials^37^. Considering that the characters of migrating cells into scaffold depend on pore sizes^37,38^ it is fascinating to speculate the GS pore size is suitable for nutrient and oxygen diffusion for BMDCs.

Some scaffolds for collagen matrices and dermis replacement including the Novosorb™ Biodegradable Temporising Matrix (BTM) or Matriderm (Medskin Solution Dr. Suwelack AG, BIllerbeck, Germany) have been already used in clinical practice^39^.

Notably, Matriderm is able to be applied to small defects with exposed tendons or bones as well as full-thickness skin defects^40,41^. However, autologous skin grafting is required on these dermal substitutes to reconstruct wounds and it is impossible to regenerate skin appendages such as hair follicles or nerve distributions in these treatments. In these respects, GS application for a lack of need of skin grafting would be promising in clinical practice for the achievement of regeneration, not scar, including skin appendages and nerves, though GS application alone appears vulnerable to infection as BTM and Matriderm. Further studies should be necessary to establish GS resistant to infection.

It is interesting to note that the periosteum is regenerated after vasculogenesis by CD105/CD106-positive BMDCs. Recently, substantial fractions of haematopoietic stem cells (HSCs) have been shown to be CD105/CD106-positive in bone marrow in mice^25^. Thus, migrating BMDCs into GS might involve HSCs that would differentiate into endothelial precursor cells. Intriguingly, HSCs-derived circulating fibrocytes have been known to contribute wound healing^42–44^. Fibrocytes are able to differentiate various cells associated with wound healing including fibroblasts. Recently, single-cell analysis of fibroblasts in wounds revealed a subset of fibrocytes differentiate into endothelial cells^45^. Hence, the fibrocytes which differentiate into endothelial cells might involve in CD105/CD106-positive BMDCs which contributed to vasculogenesis by GS application. But such analyses are beyond the scope of this study, taking into account that the specific markers for differentiating endothelial cells from fibrocytes have not been determined^44,45^.

One of the next questions is the source of BMDCs supplying to the surface of the bone cortex. Considering that 3D imaging of the calvaria bone in mice recently determined that microvessels have networks from the dura mater to the periosteum through calvaria bone, it appears that GS allowed the migration of HSCs from the exposed bone cortex^46^.

Simultaneously, vasculogenesis by BMDCs is facilitated in degrading GS in the dermis and subcutaneous tissues. Our results suggested that endothelial cells, pericytes, and smooth muscle cells, all of which contributed to vasculogenesis, were of BMDC origin in GS (+) rats, while immunostaining using anti-SMA antibodies revealed myofibroblast assembly in GS (−) rats^47^. In fact, BMDCs did not differentiate into vascular cells but were myofibroblasts in GS (−) rats. Consistently, a sort of myofibroblast has been shown to be the myeloid lineage^45^. Myofibroblast persistence leads to excessive scarring, whereas the transition to fibroblasts forms scars^47^. Recently, prevention of *Engrailed*-1 activation in fibroblasts enabled skin tissue regeneration^48^. Therefore, it is fascinating to hypothesize that GS prevents *Engrailed*-1 activation in fibroblasts because GS blocks mechanotransduction, which induces *Engrailed*-1, although further studies are needed.

Conversely, regenerated organs in the skin, such as hair follicles and nerves, were provided not by BMDCs but by local cells. In mice, de novo hair follicles observed in the wound centre were reported, which was termed wound-induced hair neogenesis (WIHN)^49^. Interestingly, WIHN is reported in rabbits^50^ as well as in sheep^51^ but not in laboratory rats^52^. Our study showed that GS application enabled WIHN in rats. Since the precise mechanism of WIHN remains unknown, our model is appropriate to pursue it beyond animal species.

This study has several limitations, one of which is that stem cells regenerating each organ, such as the periosteum, dermis, epidermis, and hair follicles, were not determined. This is because of the limitations of genetic rat models, unlike genetic-engineered mice, in which the descendants of local stem cells are capable of chasing^53^.

In conclusion, we opened up new possibilities for scarless wound healing in deep wounds with periosteal defects by applying GS as scaffolds, which did not require highly invasive treatments, such as surgery, to patients. Taking into account long experience in using GS as a haematostatic material, “repositioning” of this material will promise an easy and smooth process of clinical use for deep wounds with periosteal defects.

## Methods

### Ethics

All animal experiments were carried out in compliance with the guidelines of Shiga University of Medical Science for the care and use of animal research and the ARRIVE guidelines (Animal Research: Reporting of In Vivo Experiments). The protocol was approved by the Committee on the Ethics of Animal Experiments of Shiga University of Medical Science (Permission Number: #2017-5-17H).

### Mice

#### Animals

Sprague‒Dawley (SD) rats and SD-Tg (CAG-EGFP) CZ-004Osb (GFP) rats (provided by Dr. M. Okabe and Dr. T. Suzuki, Japan SLC, Inc., Kyoto, Japan) were used in this study. GFP rats were mated with SD rats, and their offspring were used as donors for bone marrow transplantation (BMT). Seven-week-old male wild-type SD rats (Japan SLC Co., Ltd., Kyoto, Japan) were used as recipients.

Animals were housed at the Research Center for Animal Life Science, Shiga University of Medical Science, and maintained in a temperature- and humidity-controlled (23 ± 1 °C, 60 ± 10%) environment. During the experimental periods, rats were given free access to water and CLEA Rodent Diet CE-2 (CLEA Japan, Inc., Tokyo, Japan).

### Bone marrow transplantation (BMT)

The recipient rats were exposed to 9 Gy total body irradiation from an X-ray source (MBR-1520R HITACHI Medico, Inc., Tokyo, Japan). Bone marrow from the femurs, tibias and humeri of the transgenic donor rats was harvested by flushing with phosphate-buffered saline (PBS). Six hours after irradiation, 1.0 × 10^8^ BMDCs were injected into the tail vein of the recipient rats. Four weeks after BMT, peripheral leukocytes from recipient rats were analysed for GFP expression by flow cytometry on a FACScant II (Becton and Dickinson, Franklin Lake, NJ, USA).

### Animal models of deep wounds with periosteal defects

Under general anaesthesia with 2% isoflurane in combination with intraperitoneal injection of edetomidine hydrochloride (0.15 mg/kg), midazolam (2 mg/kg), and butorphanol tartrate (2.5 mg/kg), a 10 mm × 10 mm (100 mm^2^) square defect of the full-thickness skin with/without the periosteum was made in the calvaria of wild-type or BMT rats. A sponge (LTL Pharma Co., Ltd, Tokyo, Japan) was applied to the wounds in one group, whereas wounds were left open in the other group. Unwounded animals were used as controls. The wounds were photographed at 0, 2, 4, and 6 w pw, and the percentage of areas of wounds to initial wound area (100 mm^2^) were measured using ImageJ software (National Institutes of Health, Bethesda, MD, USA). Four rats were prepared in each group, and the experiments were repeated twice.

### Histology and immunohistochemistry

After exsanguination followed by transcardial perfusion with 4% PFA in 0.1 M PBS, the calvaria were decalcified with 10% EDTA for 24 h at 4 °C. Sections of the calvaria were prepared with a cryostat at 7 μm thickness for staining with haematoxylin and eosin reagent. For immunohistochemistry, the sections were treated with 5% normal goat serum for 1 h at 25 °C and incubated with primary antibodies overnight at 4 °C, followed by incubation with secondary antibodies for 2 h at 25 °C.

The primary antibodies used in this study were as follows: anti-smooth actin (1:200, ab5694, Abcam plc, Cambridge, UK), anti-CD31 (1:50, ab281583, Abcam plc, Cambridge, UK), anti-NG2 (1:100, 55027-1-AP, Proteintech Group Inc, Rosemont, IL, USA), anti-CD105 (1: 200, bs-0579R, BIOSS Inc, Woburn, MA, USA), anti-CD106 (1:200, 39036, Cell Signaling Technology Inc, Danvers, MA, USA), anti-Lef1 (1: 200, 2230, Cell Signaling Technology Inc, Danvers, MA, USA), anti-PGP9.5 (1: 800, RA12103, Neuromic Inc, Edina, MN, USA), and anti-POSTN (1: 200, ab215199, Abcam plc, Cambridge, UK). The secondary antibody used was goat anti-rabbit Alexa 555 (1:400, A27039, Thermo Fisher Scientific, MA, USA).

Sections for immunofluorescence were mounted with Vector Shield using 4′-6-diamidino-2-phenylindole (DAPI) (Vector Laboratories, Burlingame, CA, USA) and photographed using a laser scanning confocal microscope (Leica TCS SP8, Leica Microsystems GmbH, Mannheim, DE).

### Analyses of the GFP+ cell population and the numbers of vessels in each regenerated layer

The percentage of GFP+ cells to total cells in the dermis, subcutaneous tissue, and periosteum was calculated as follows: The square areas with 200 μm × 200 μm within 400 μm or 800 μm below the basal layer of the epidermis were selected as the dermis or the subcutaneous tissue, respectively. Rectangular areas of 200 μm × 171.9 μm to 18.34 μm, depending on the thickness of the periosteum, were selected as the periosteum region. The numbers of GFP+ cells, total cells and vessels were counted per area at 4 w and 6 w pw in every section of wounds with periosteal defects in GS (−) or GS (+) rats. The experiments were repeated twice.

### Statistical analyses

Data were evaluated using *t* tests to analyse differences between two groups or using Kruskal Wallis test to analyse differences among more than the three groups. Data are expressed as the mean ± SE. P < 0.05 was considered significant.

## Supplementary Information

Supplementary Information.

## Data Availability

The datasets used and/or analysed during the current study available from the corresponding author J. O. (email: jokano@belle.shiga-med.ac.jp) on reasonable request.
