# Supplementary material for: Bone marrow-derived vasculogenesis leads to scarless regeneration in deep wounds with periosteal defects
Source: Sci Rep. 2022 Nov 29;12:20589. doi: 10.1038/s41598-022-24957-1 (PMC9708684; doi:10.1038/s41598-022-24957-1)
Supplement: Supplementary file 1 — Supplementary Information. [file 41598_2022_24957_MOESM1_ESM.pdf]

%GFP	4weeks after BMT
GS-	87.40±1.12
GS+	88.47±1.65
control	89.80±2.64

Supplementary Table. 1. Chimaerism analyses of rats after bone marrow transplantation (BMT). Bone marrow transplantation completely replaced for peripheral blood cell from donor origin at 4 weeks after BMT. The average values indicate ± SE. There is no significant difference between GS-/+ and control group. n=4 for each. The experiments were repeated twice.

	dermis						periosteum			
	GFP+SMA+		GFP+CD31+		GFP+NG2+		GFP+CD105+		GFP+CD106+	
	GS-	GS+	GS-	GS+	GS-	GS+	GS-	GS+	GS-	GS+
2 w pw	-	-	-	±	-	-	-	-	-	-
4 w pw	-	++	-	±	-	+	-	++	-	++
6 w pw	±	++	±	±	±	+	±	±	±	±

Supplementary Table. 2.

Quantitative analyses of BMDCs contribution to regenerated vessels or periosteum in GS (-) or GS (+) rats. The numbers of SMA-, CD31-, NG2-, CD105- or CD-106 positive cells with GFP-positivity were counted in five fields of view in the dermis (200 μm x 200 μm) or the periosteum (200 μm x 171.9 μm to 18.34 μm, depending on the thickness of the periosteum) per GS (-) or GS (+) rats, respectively. Each symbol indicates the following: -, 0 to 5 in each field; ±, 5 to 15 in each field; +, > 15 in each field. n=4 for each. The experiments were repeated twice.

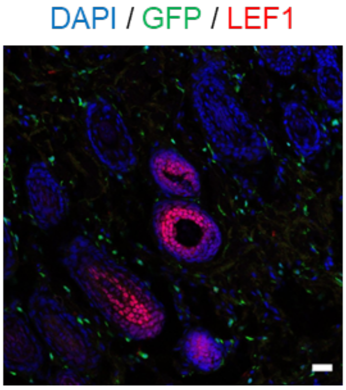

Supplementary Fig. 1. Hair follicles in no wounded areas express LEF1. Scale bars, 30 µm
